# Systems biology for crop improvement

**DOI:** 10.1002/tpg2.20098

**Published:** 2021-05-05

**Authors:** Lekha T. Pazhamala, Himabindu Kudapa, Wolfram Weckwerth, A. Harvey Millar, Rajeev K. Varshney

**Affiliations:** ^1^ Center of Excellence in Genomics & Systems Biology International Crops Research Institute for the Semi‐Arid Tropics (ICRISAT) Patancheru Hyderabad 502 324 India; ^2^ Department of Ecogenomics and Systems Biology University of Vienna Vienna Austria; ^3^ Vienna Metabolomics Center University of Vienna Vienna Austria; ^4^ ARC Centre of Excellence in Plant Energy Biology and School of Molecular Sciences The University of Western Australia Perth WA Australia; ^5^ State Agricultural Biotechnology Centre Crop Research Innovation Centre, Food Futures Institute, Murdoch University Murdoch WA Australia

## Abstract

In recent years, generation of large‐scale data from genome, transcriptome, proteome, metabolome, epigenome, and others, has become routine in several plant species. Most of these datasets in different crop species, however, were studied independently and as a result, full insight could not be gained on the molecular basis of complex traits and biological networks. A systems biology approach involving integration of multiple omics data, modeling, and prediction of the cellular functions is required to understand the flow of biological information that underlies complex traits. In this context, systems biology with multiomics data integration is crucial and allows a holistic understanding of the dynamic system with the different levels of biological organization interacting with external environment for a phenotypic expression. Here, we present recent progress made in the area of various omics studies—integrative and systems biology approaches with a special focus on application to crop improvement. We have also discussed the challenges and opportunities in multiomics data integration, modeling, and understanding of the biology of complex traits underpinning yield and stress tolerance in major cereals and legumes.

## Core Ideas

- Multiomics data enable understanding and prediction of plant phenotype with higher precision.
- Systems biology approach involves multiomics data integration and modeling.
- Integrative systems biology aids better prediction of cellular functions of complex traits.
- Advancements, challenges, and opportunities in systems biology research are reviewed.
- Prospects of systems biology to crop improvement are presented.

**Abbreviations**
BPH: brown planthopper
GEA: gene expression atlas
GWAS: genome‐wide association study
NGS: next‐generation sequencing
QTL: quantitative trait loci
SNP: single nucleotide polymorphism
SV: structural variation.

## 1 INTRODUCTION

Crops such as cereals and legumes play an important role in human diet by providing necessary calories, proteins, essential amino acids, and minerals. Over the last 100 yr of extensive breeding, crop varieties have been developed for higher yields. However, because of increasing global population, there is an urgent need to double the yield by the year 2050. This increase in production and productivity is challenging considering the current environmental constraints and rapidly depleting natural resources. Model plants, such as Arabidopsis (*Arabidopsis thaliana* (L.) Heynh.) and rice (*Oryza sativa* L.), have been extensively studied for comprehensive understanding of plant genetics, genomics, and defining the function of specific genes. This is essential for leveraging genomics for breeding a new generation of climate‐ready crops to produce surplus food that is high in nutrients. Toward this, a genomics‐assisted breeding (Varshney et al., 2005) approach can greatly accelerate the existing crop improvement programs. Translation or transfer of genetic information gained from one species to another was quite limited before the genomics era, mainly because of a lack of suitable knowledge in genomic information and systems biology.

The advent of next‐generation sequencing (NGS) technologies has revolutionized and increased the pace of generation of genomics and transcriptomics data that has led to new era of the ‘big data.’ Several NGS platforms, such as Illumina's MiSeq/HiSeq; Roche's 454/FLX; ABI/Life Technologies’ SOLiD; Invitrogen's Ion Proton, have led to the sequencing of genomes and transcriptomes for a number of plant species (Barutcu et al., 2015; van Dijk et al., 2018). Third‐generation sequencing technologies such as single‐molecule sequencing by Helicos Biosciences (HeliScope), single molecule real‐time sequencing by Pacific Biosciences (PacBio), and Nanopore sequencing by Oxford Nanopore Technologies accelerated generation of large‐scale sequencing data (Giani et al., 2020). This has dramatically changed the sequencing scenarios and led to the development of high‐quality genome assemblies in several crop plants including complex and large‐sized genomes. The big data from omics experiments are analyzed with advanced software programs and analytical methods to understand the complexity of biological systems. This new area of research has come to be known as either integrative biology or systems biology. Integrative biology focuses on combining omics layers to build insight based on the Aristotelian principle that the whole is greater than the sum of the parts. Systems biology focuses on combining omics layers to build models that explain system behavior and that have predictive power to propose the outcome of mutation or modification of specific biological steps. Furthermore, systems biology has applications in dissecting complex agronomic traits and in the model‐based prediction of phenotypes in different conditions (Lavarenne et al., 2018). The development of user‐friendly pipelines and bioinformatic tools to analyze the big data generated by omics approaches can further facilitate breeding programs both through enhanced selection tools and through more sophisticated design of crossing programs or stacking of gene modifications.

**Core Ideas**

- Multiomics data enable understanding and prediction of plant phenotype with higher precision.
- Systems biology approach involves multiomics data integration and modeling.
- Integrative systems biology aids better prediction of cellular functions of complex traits.
- Advancements, challenges, and opportunities in systems biology research are reviewed.
- Prospects of systems biology to crop improvement are presented.

**FIGURE 1:**
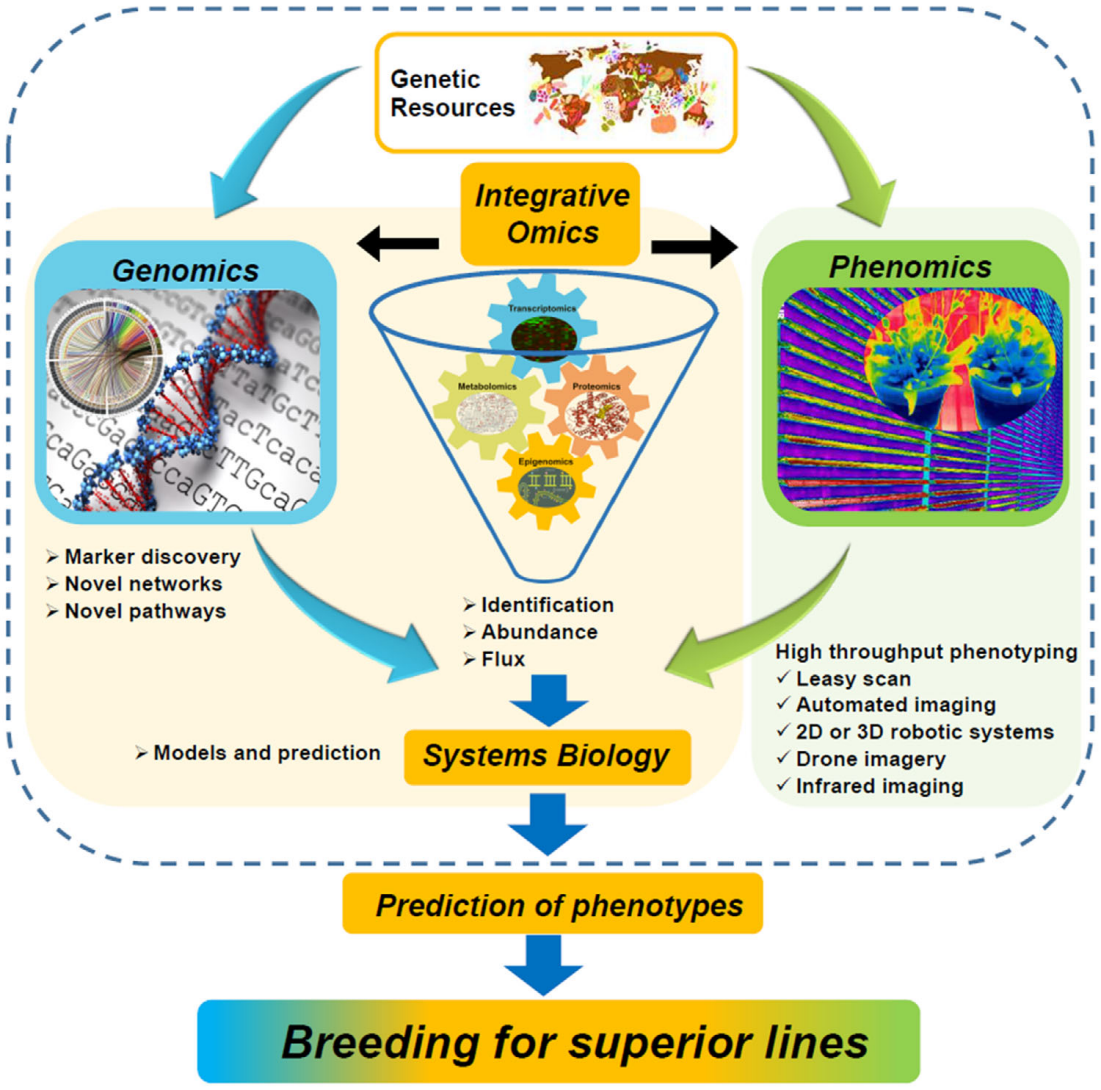
An overview of the integrated omics in genetics and breeding for crop improvement. ‘Omics revolution’ is an integrated comprehensive omics approach combining genomics, transcriptomics, proteomics, metabolomics, epigenomics, and other breeding tools for advancement of systemic sciences and to accelerate genomics‐enabled, next‐generation breeding

An overview of current omics resources, multiomics data integration, and systems biology approaches each with a focus on their applications in plant research and breeding has been discussed in the present review (Figure 1). We highlight existing and emerging approaches that contribute to our understanding of the biology of complex traits and holistic improvement of yield together with tolerance and resistance to abiotic and biotic stresses. Furthermore, the prospects and challenges facing multiomics data integration, modeling, and systems‐level analyses, particularly with the fast‐emerging omics technologies, have been discussed. The thoughts presented in this review provide insights on applications of integrated multiple omics research and systems biology for crop improvement.

## 2 RAPIDLY EVOLVING OMICS APPROACHES

High‐throughput technologies have revolutionized plant research through the study of a whole set of biological entities, including DNA, RNA, proteins, metabolites, and others, of a given species and have been noteworthy. This high‐throughput measurement has led to an array of approaches carrying the omics suffix such as genomics, pangenomics, transcriptomics, proteomics, metabolomics, epigenomics, and, more recently, single‐cell omics, phenomics, and QTL‐omics. These approaches are now integrated across multiple omics layers, providing an opportunity to understand the flow of information that underlies trait biology. In the following sections, we will be discussing these rapidly evolving omics approaches for crop improvement.

### 2.1 Genomics and pangenomics

#### 2.1.1 Genome sequencing and resequencing

Understanding the structure, organization, and dynamics of genomes in plant species can provide insights into how genes have been adapted or altered by natural and artificial selection in response to environmental constraints. Studies have demonstrated the potential use of the adapted and manipulated genes for crop improvement not only within a species but also the use of such genes across species (Kawashima et al., 2016). Toward accomplishing these goals, many plant genomes have been sequenced (Kersey, 2019). Earlier, only a few plant species with relatively compact genomes, and known as ‘models,’ were sequenced to understand genome architecture because of the high cost of sequencing and limited expertise. As a result, genome sequence information for *Arabidopsis thaliana* (L.) Heynh., rice (*Oryza sativa* L.), black cottonwood (*Populus trichophora* Torr. & A. Gray), grapevine (*Vitis vinifera* L.), and maize (*Zea mays* L.) were the first plant genomes generated. Later, legumes such as soybean [*Glycine max* (L.) Merr.], pigeonpea [*Cajanus cajan* (L.) Huth], and chickpea (*Cicer arietinum* L.) genomes were sequenced (see Varshney et al., 2015).

Evolution in NGS and third‐generation sequencing technologies has resulted in ever‐increasing throughput and reduced sequencing costs. As a result, more than 600 complete plant genome assemblies are available in public repositories (Kersey, 2019) and many more are being sequenced (http://www.onekp.com/). The genome sequence information generated through high‐throughput sequencing of germplasm collection is also enabling the simultaneous discovery and sequencing of thousands of genetic markers across whole genomes (Varshney et al., 2019a). These new sequencing tools are also valuable for the validation and assessment of genetic markers in populations. Further, it has been possible now to identify all the genes in a plant, which would in turn help understand the genetic properties as well as networks that contribute to develop superior crop varieties.

In addition to information made available from the genome sequence of cultivars, characterization of genetic diversity present in wild crop relatives and landraces conserved in gene banks are a valuable source of novel genes that could enhance yield and resistance to abiotic and biotic stresses. In this context, resequencing efforts of large‐scale germplasm collections have become important. One such example is the 3,010 diverse Asian cultivated rice genomes from the 3,000 Rice Genomes Project (Wang et al., 2018). The study identified 29 million single nucleotide polymorphisms (SNPs), 2.4 million small indels, and over 90,000 structural variations (SVs) that contribute to within‐ and between‐population variation. This study highlighted the genetic diversity that exists in rice germplasm repositories with agriculturally relevant phenotypes. Further the study demonstrated the use of identified SNPs in trait mapping analysis for the highly heritable traits, grain length, grain width, and bacterial blight resistance in rice (Wang et al., 2018). Another example of maize reported sequencing of 278 maize inbred lines and demonstrated extensive variation in SNPs (27 million), indels (287,504), and copy number variations that can potentially be used as a selection index in future maize breeding programs (Jiao et al., 2012). The study showed that modern breeding has introduced dynamic genetic changes into the maize genome, and further artificial selection has affected thousands of targets including genes and nongenetic regions leading to a reduction in nucleotide diversity and an increase in the proportion of rare alleles (Jiao et al., 2012). In the case of dryland cereals, for example, resequencing of 44 sorghum [*Sorghum bicolor* (L.) Moench] lines representing the primary gene pool and spanning dimensions of geographic origin, end use, and taxonomic group resulted in identification of 8 million SNPs, 1.9 million indels, and specific gene loss and gain events for use in sorghum improvement (Mace et al., 2013). This study on sorghum presented that a large untapped pool of diversity exists not only in races of sorghum but also in the allopatric Asian species *S. propinquum* (Kunth) Hitchc. A strong racial structure and complex domestication events were observed with in the accessions studied. Similarly, in pearl millet [*Cenchrus americanus* (L.) Morrone], resequencing of 994 lines resulted in identification of more than 29 million SNPs and 3 million indels for better understanding of trait variation and accelerating genetic improvement (Varshney et al., 2017b). This study highlighted the application of resequencing data to understand the population structure, genetic diversity, and domestication of pearl millet. Further genomic prediction was employed to predict pearl millet hybrid performance and genome‐wide association study (GWAS) predicted yield‐associated traits in both irrigated and drought conditions.

In legumes, soybean for instance, resequencing of 302 wild and cultivated soybean accessions identified 9 million SNPs and 876,799 indels, providing genes related to domestication and resources for genomics‐enabled crop improvement (Zhou et al., 2015). In a different study, 17 wild and 14 cultivated soybean genomes have been resequenced (Lam et al., 2010), thus revealing patterns of genetic variation between wild and cultivated soybeans. This study identified greater allelic diversity in wild soybean and a set of 205,614 SNPs for use in quantitative trait loci (QTL) mapping and association studies. Very recently, Valliyodan et al. (2021) analyzed genetic diversity and structure from the resequencing of 481 diverse soybean accessions, comprising 52 wild selections and 429 cultivated varieties (landraces and elites). This study identified evidence of distinct, mostly independent selection of lineages by particular geographic location. Recently, we have also undertaken the sequencing and phenotyping of thousands of global composite collection of chickpea genomes as part of the 3,000 Chickpea Genome Sequencing Initiative. Under this initiative, resequencing of 429 chickpeas sampled from 45 countries identified a map of 4.97 million SNPs and GWAS identified 262 markers and several candidate genes for 13 different traits associated with drought and heat tolerance mechanisms (Varshney et al., 2019b). Similar efforts were carried out in pigeonpea, where resequencing of 292 pigeonpea accessions resulted in identification of 15.1 million SNPs and 2.1 million indels. This study revealed genomic regions associated with domestication and markers linked with key traits such as flowering time control, seed development, and pod dehiscence (Varshney et al., 2017a). In brief, the sequencing and resequencing studies in several crop species demonstrated the use of genomes and SNPs for trait mapping analyses. Such studies are expected to guide and accelerate crop breeding by identifying genetic variation that will be useful in breeding efforts in different crops and future sustainable agriculture.

#### 2.1.2 Pangenomics

High‐throughput resequencing technologies have been employed in several crops with an aim to explore genomic diversity and to uncover molecular basis of important agronomic traits. However, in all these resequencing studies, characterization of the genetic variants depends on high levels of sequence similarity to map the short reads onto the reference genome, which may miss highly polymorphic regions and regions that are not present in the reference genome (Zhou et al., 2015). Therefore, with an objective to capture all possible variations in a given germplasm collection of a particular species, pangenomics studies have been conducted in several species (see Khan et al., 2020).

In rice, a pangenome of cultivated rice–wild rice (*O. sativa* *‐O. rufipogon* Griff.) species complex through deep sequencing and de novo assembly of 66 divergent accessions was constructed (Zhao et al., 2018). In this study, intergenomic comparisons identified 23 million sequence variants in the rice genome. In maize, pangenome was characterized using 503 inbred lines and loci associated with plant developmental transitions, fitness, and adaptation traits were identified (Hirsch et al., 2014). Pangenome in wheat (*Triticum aestivum* L.) was built using 18 cultivars, which resulted in identification of 128,656 genes of which 64.3% were core genes while the remainder are variable and display presence–absence variation (Montenegro et al., 2017). In legumes, pangenome was established in soybean using seven phylogenetically and geographically representative accessions of wild soybean (*G. soja* Siebold & Zucc.), the wild relative of cultivated soybean. Analysis of the soybean pangenome identified 80% core genes. Furthermore, intergenomic comparisons identified genes associated with biotic resistance, seed composition, flowering and maturity time, and others (Li et al., 2014). Recently, another study reported pangenome of 26 representative wild and cultivated soybean selected from 2,898 globally collected soybean germplasm in terms of phylogenetic relationships and geographic distributions (Y. Liu et al., 2020). The pangenome identified large SVs and gene fusion events in soybean. The SVs identified in the study were linked to gene expression and important agronomic traits such as seed luster, seed coat pigmentation, and iron deficiency chlorosis. In the case of pigeonpea, the first pangenome based on 89 accessions was reported (Zhao et al., 2020). The study identified genes associated with important agronomic traits such as seed weight, self‐fertilization, and response to disease in pigeonpea. Pangenome using eight high‐quality rapeseed (*Brassica napus* L.) genomes revealed architecture and ecotype differentiation (Song et al., 2020).

Pangenome provides in‐depth dissection of dispensable as well as species‐specific genes identified mostly from cultivated gene pool. In order to achieve complete genetic repertoire of a given crop, diverse gene stock coming from wild species is imperative. Here comes in the concept of a super‐pangenome, which represents a complete genomic variation repertoire by combining different pangenomes from all the species within a given genus (Khan et al., 2020). Deployment of the super‐pangenome concept by integrating the wild side of a species with diverse genetic stock will help in tapping genetic diversity from wild species for accelerating crop improvement.

These studies provide useful insights into the genetic variability, population structure, and diversity of important crop species that could be used for crop improvement programs (Tao et al., 2019). As sequencing and resequencing costs are continuously decreasing, sequence‐based allele discovery has become more prevalent. Systematic application of genome‐wide sequence information in support of crop improvement as translational genomics for agriculture will accelerate the precision of crop breeding cycle (Bohra et al., 2020; Varshney et al., 2015). The genomic resources developed in crop species will facilitate the dissection of complex traits and identification and exploitation of SNPs and SVs associated with traits of interest (Thudi et al., 2021). Furthermore, knowledge of resequencing and pangenomes and super‐pangenomes would provide information on an untapped pool of diversity for easy access in breeding resulting in genetic improvement of crop species to meet future food demands.

#### 2.1.3 Genome sequence variations, gene prediction, and functional inferences

The major challenge for crop improvement is increasing the productivity while reducing yield losses that result from various biotic and abiotic stresses under climate change scenarios (Palit et al., 2020b). Therefore, the major aim of genomic studies in crop plants has been the identification of key regulatory genes and the active pathways associated with plant architecture and crop yields. Intensive sequence‐level characterization of a chromosomal region and cloning reveals the presence of novel genes of unknown function (Jaganathan et al., 2020). Unique alleles, the makeup of alleles, variation in gene expression, signature sequences, among other, are important contributors to phenotypic diversity within and between species. Therefore, a 5G breeding approach for bringing in the much‐needed disruptive changes to crop improvement has been proposed by Varshney et al. (2020). Unlimited genomics resources, such as SNPs and genome‐wide SVs, are made available through sequencing and resequencing of diverse germplasm in different crops (Thudi et al., 2021). These resources would facilitate genotyping the landraces and breeding material for identification of candidate genes and diagnostic markers for the traits of interest in crop species. For example, genome sequencing of rice subspecies SN265 (*O. sativa* L. subsp. *japonica* Kato), R99 (*O. sativa* L. subsp. *indica* Kato), and resequencing of a total of 151 recombinant inbred lines generated from the cross between these parents revealed 1.7 million SNPs. Analysis of the data revealed yield and quality‐associated loci and the involvement of a candidate gene, *DEP1*, in determining panicle length (X. Li et al., 2018). Similarly, the genome assembly of a maize small‐kernel inbred line derived from tropical landrace provides insights into 80,614 polymorphic SVs across 521 diverse lines (N. Yang et al., 2019). Further dissection through map‐based cloning of a major effect quantitative trait locus controlling kernel weight revealed the underlying candidate gene, *ZmBARELY ANY MERISTEM1d*, that provides a target for increasing maize yields.

In the case of legumes, for example, the genome of cultivated peanut (*Arachis hypogaea* L.) provided insight into legume karyotypes, polyploid evolution, and crop domestication. The QTL for seed size and testa color have been mapped to the peanut reference genome (Zhuang et al., 2019). Comprehensive analysis of resequencing data has enhanced our understanding of complex traits and provided candidate genes for several agronomic and disease‐resistance traits for use in crop breeding (Varshney et al., 2019a). For instance, resequencing of 302 wild and cultivated soybean accessions revealed 230 selective sweeps and 162 selected copy number variants and correlated 96 of the 230 selected regions with oil QTL and fatty acid biosynthesis genes (Zhou et al., 2015). Similarly, in common bean (*Phaseolus vulgaris* L.), GWAS on a panel of 192 genotypes revealed candidate genes for flowering time variation (Raggi et al., 2019). In chickpea, using whole‐genome resequencing data from 132 chickpea lines, GWAS identified four genetic regions containing 38 SNPs significantly associated with yield and yield‐related traits (Y. Li et al., 2018). Furthermore, increase in prediction accuracy has been demonstrated in this study by incorporating results from GWAS in genomic selection. In the case of pigeonpea, markers for seed protein content were developed from whole‐genome resequencing data from four pigeonpea lines demonstrating the potential of genomics (Obala et al., 2019).

With copious amount of genomics information in model and crop species, comparative functional analysis of genomics data facilitates gaining new insights toward exploring new genes and traits for potential application in crop improvement. For example, X. Yang et al. (2019) demonstrated the use of comparative genomics in evolutionary mechanisms and gene function in crassulacean acid metabolism plants. Similarly, based on genome information of pigeonpea, Kawashima et al. (2016) reported cloning of a *Phakopsora pachyrhizi* resistance gene *CcRpp1* (*Cajanus cajan* resistance against *Phakopsora pachyrhizi* 1) from pigeonpea and showed that *CcRpp1* confers resistance to *P. pachyrhizi* (causing soybean rust) in soybean. In summary, genomics resources have an enormous scope in modernizing crop breeding programs to deliver next generation varieties.

### 2.2 Transcriptomics

Transcriptomics explains the conceptual changes encompassing not only in the genome itself but also the process by which the information contained in the genome is used by the cell and to discover the flow of biological information from the genome to the cell. To begin with, efforts have been focused on the development of complementary DNA libraries, generation of expressed sequence tags, gene expression analysis, and the *in silico* mining of functional information from expressed sequence tags data sets even before genome sequences were available (Varshney et al., 2009). Initial gene expression studies relied on low‐throughput methods. However, a RNA sequencing approach provides higher coverage and greater resolution of transcriptome dynamics when compared with Sanger sequencing and microarray‐based methods (Garg et al., 2019).

Transcriptome sequencing is being widely used in studying plant responses to various stresses as well as its growth and development. In addition, transcriptome sequencing has been applied for various functional genomics purposes such as gene expression profiling, genome annotation, and the discovery of noncoding RNA (Morozova & Marra, 2008). Broad availability of NGS technologies led to a paradigm shift in molecular breeding of crop species and are expected to directly detect epigenetic modifications on native DNA and to allow whole‐transcript sequencing without the need for genome assembly (van Dijk et al., 2018). Several transcriptome assemblies have been generated for major crops such as rice (Tian et al., 2015), wheat (Jia et al., 2018), and maize (Zhang et al., 2019a) to aid in elucidating molecular regulation of candidate genes for different traits at different stages of growth and development of the plant under stress and control conditions. In legumes, for example, hybrid comprehensive assemblies were generated in the case of pigeonpea (Kudapa et al., 2012) and chickpea (Kudapa et al., 2014) by analyzing sequencing data from three different platforms (Sanger, FLX/454, and Illumina). In a different study, transcriptome analysis under elevated CO_2_ concentrations identified stress responsive candidate genes and pathways mainly involved in sugar and starch metabolism, chlorophyll, and secondary metabolites biosynthesis (Palit et al., 2020a). Gene expression profiling data from developed transcriptome assemblies enabled identification of candidate genes associated with different traits of interest and stress response.

In addition to identifying candidate genes for the traits of interest and stress response, understanding how underlying genome information translates into specific phenotypes at key developmental stages is crucial. For this, information on gene expression patterns across different plant developmental stages and organs covering entire plant life cycle is required. Gene expression atlases (GEAs) allow a thorough survey of the entire transcriptional landscape, revealing genome‐wide gene activity in different tissues of several model and crop plants. In rice, GEA was developed from 39 tissues collected throughout the life cycle of the rice plant from two cultivars, Zhenshan 97 and Minghui 63 (L. Wang et al., 2010). This study provided a versatile resource for associating transcriptomics to the developmental process and understanding the regulatory process by tracing the expression profiles of individual genes. Similarly, in maize, 18 representative maize tissues capturing important aspects of maize development were targeted for GEA resulting in identification and characterization of genes and pathways underlying plant growth and development (Sekhon et al., 2013). In legumes, for example, the RNA Seq‐Atlas in soybean provided a record of high‐resolution gene expression in a set of 14 diverse tissues and identification of candidate genes involved in seed development, nodule formation, and seed filling (Severin et al., 2010). Similarly, in chickpea, 27 tissues from five major developmental stages were used to construct a comprehensive *Cicer arietinum* Gene Expression Atlas (Kudapa et al., 2018), which identified significant differences in gene expression patterns contributing to the process of flowering, nodulation, seed and root development. In pigeonpea, 30 tissues representing developmental stages from germination to senescence were used to generate a *Cajanus cajan* Gene Expression Atlas (Pazhamala et al., 2017), which provided candidate genes involved in specific developmental processes and to understand the well‐orchestrated growth and developmental process in pigeonpea. Similarly, *Arachis hypogaea* (groundnut) Gene Expression Atlas has been very useful to investigate complex regulatory networks, namely gravitropism and photomorphogenesis, seed development, allergens, and oil biosynthesis in groundnut (Sinha et al., 2020). These transcriptomic resources should be able to provide insights into the molecular mechanisms of growth and development, high yields, stress responses among many other traits, ultimately assisting in development of improved crop varieties.

### 2.3 Proteomics

Transcriptomes ultimately come to fruition through translation to build proteins that, in combination, form the complex proteomes of different tissue types and different stages of plant development. The correlation of abundance of transcripts and proteins have been analyzed in many plant systems and notably during germination, seed development, and responses to stress. This necessitates the direct study of proteins to fully understand gene expression. The use of mass spectrometry for protein identification and protein relative or absolute quantitation, referred to as proteomics, enables the study of large sets of cellular proteins that constitutes these proteomes. Proteomics approaches have transitioned from being descriptive to become highly useful for data validation and integration with other omics approaches, providing information on biological processes and stress tolerance mechanisms that can be applied in crop breeding programs (J. Hu et al., 2015). Data‐dependent ‘shotgun’ proteome sampling strategies enable large datasets of relatively quantified differences between crop varieties to be assessed but are typically limited to a small number of comparisons. Meanwhile, selected reaction‐monitoring strategies allows targeted quantitation of known proteins of interest over much larger sample sets allowing whole recombinant inbred line, near‐isogenic line, and double‐haploid populations to be screened for proteins of interest and their abundances (Jacoby et al., 2013)

The plant proteome undergoes significant changes because of the activation of stress‐responsive pathways when subjected to biotic and abiotic stress. The proteins that are known to have significant involvement in abiotic stress response include heat‐shock proteins, late embryogenesis abundant proteins, kinases and phosphatases, redox enzymes, secondary metabolism enzymes, osmolyte biosynthetic enzymes, photosynthesis, and carbon metabolism‐related and enzymatic reactive oxygen species scavengers (see Hossain & Komatsu, 2012). Posttranslational modifications of proteins are also essential features of plant response to environment and are critical for plant phenotypes associated with stress tolerances (Millar et al., 2019). Protein abundance itself is only a proxy for function, rate of protein synthesis and degradation, age of proteins, and age‐associated features of protein function is also critical to defining proteomes (Nelson & Millar, 2015). Several proteome maps have been developed in the model plants and crops, for instance, Arabidopsis (Baerenfeller et al., 2008), rice (Helmy et al., 2011), wheat (Duncan et al., 2017), barrel clover (*Medicago truncatula* Gaertn.), *Lotus japonicus* (Regel) K. Larsen, and soybean (see Ramalingam et al., 2015). Recently, proteomic analysis in three rice cultivars identified over 4,900 proteins and 1,309 differentially expressed proteins (Zhang et al., 2019b). This study identified eight genes encoding various metabolic proteins involved in brown planthopper (BPH) resistance in rice. Further, the study reported activation of the two‐component response regulator protein (ORR22) that is crucial in early signal transduction in the resistance response against BPH through sustained promotion of salicylic acid. Also, key enzymes‐lipoxygenases, dirigent proteins, and Ent‐cassa‐12,15‐diene synthase (OsDTC1) in inheritable resistance against BPH were identified for use in breeding BPH‐resistant rice cultivars (Zhang et al., 2019b). In maize, proteomic analysis under CO_2_–enriched conditions resulted in identification of changes in protein abundance that were correlated to yield and related traits (Maurya et al., 2020). Reduced malondialdehyde content and antioxidant and antioxidative enzymes levels were observed in response to high CO_2_. Further, more abundance of proteins related to Calvin cycle, protein synthesis assembly and degradation, defense, and redox homeostasis contributed to better growth and yield in elevated CO_2_ was reported (Maurya et al., 2020). In legumes, for example, soybean, proteomics together with physiological and biochemical analysis led to identification of cross‐tolerance mechanisms in response to heat and water stresses (Katam et al., 2020). The study reported elevated activities in antioxidant enzymes, such as increased ascorbate peroxidase enzyme, which restored oxidation levels and sustained soybean plants during stress. In addition, proteins such as MED37C, a probable mediator of RNA polymerase transcription II, were elevated in response to combined heat stress and water stress levels (Katam et al., 2020).

### 2.4 Metabolomics

After the establishment of transcriptomic and proteomic profiles, the next functional genomics challenge is the function of enzymes that build the complex milieu of primary and secondary photosynthetic catabolites as well as the sophisticated array of biosynthetic products that make up the metabolome. Metabolomics is the study of these small molecules in plants and the dynamic changes in their abundance on diurnal, development, and stress‐responsive timescales (Fiehn et al., 2000; Weckwerth, 2003). Metabolomics encompasses a rapidly changing suite of technologies including mass spectrometry, multiple types of spectroscopy, and nuclear magnetic resonance spectroscopy. Metabolite pool sizes of major metabolites are of value in assessing the metabolome (Nunes‐Nesi et al., 2019) but it is increasingly recognized that it is the flux through these major metabolite pools that contribute significantly to plant growth and development and stress biology (Moreira et al., 2019). Identifying rarer compounds including byproducts of signal transduction molecules, stress metabolism, and molecules that are part of plant acclimation process (Larrainzar et al., 2009) will be critical in breeding for adaptive metabolic traits. Metabolic compounds identified can also be further studied by correlation with transcriptome and proteome expression patterns. The cascading effects of gene expression on different levels of biological organization lead to the phenotype. Metabolites can directly influence the cellular physiology, thus closest to the phenotype (Guijas et al., 2018) but not directly related to the genome (Redestig & Costa, 2011). The profiling of metabolites corresponding to those of the transcripts under a specified condition or in a particular genotype allows an understanding of developmental processes and plant response to external stimuli or metabolism. This approach has been demonstrated in crops such as rice (C. Hu et al., 2014), soybean (Komatsu et al., 2011), and common bean (Hernández et al., 2007), in which up to 100 known metabolites have been shown to change in abundance based on geographic origin of the seeds and in response to flooding or nutrient limitation. Metabolic fingerprinting is used to identify metabolic signatures associated with stress responses without quantifying or identifying metabolites, for example, nuclear‐magnetic‐resonance‐based approach for metabolic fingerprinting of 21 grass and legume cultivars (Bertram et al., 2010). Further, considering plant metabolome as the readout of their physiological status, metabolite‐based GWAS has been utilized to dissect the genetic and biochemical bases of metabolism in crop plants (Luo, 2015). Metabolite‐based GWAS has established a strong genotype–metabolite associations in maize and rice (Chen et al., 2014; Dong et al., 2014; Matsuda et al., 2012; Wen et al., 2014). Metabolomics study would provide important insights that can serve as a basis for future crop improvement via metabolic engineering (see Kusano & Saito, 2012).

### 2.5 Epigenomics

Epigenomics is the study of all epigenetic modifications in a cell. Epigenetic changes are heritable changes in gene expression and cellular functions as a result of DNA methylation, histone modifications, and biogenesis of noncoding RNAs without altering the underlying DNA sequence. Several studies in recent years helped to better understand the role of the epigenome in plant biotic and abiotic stress responses (see Agarwal et al., 2020). Further, comprehensive epigenomic studies of plant populations to correlate genotype–epigenotype–phenotype, and also the study of methyl QTL or epiGWAS will widen the understanding of mechanisms as well as functions of regulatory pathways in plant genomes (Yadav et al., 2018). Following the identification of key epigenetic regulators, epigenomics toward systems biology is needed to understand the dynamic and complex functional relationships at the plant systems level. Engineering epigenomes and epigenome‐based predictive models will further accelerate molecular breeding programs for crop improvement.

### 2.6 Single‐cell omics

Most of the studies undertaken to understand plant biology were at the level of tissue, organ, or complete plant, which unraveled the biological activities and the genes involved. However, these studies could obscure the specific biological function of the individual cells or low‐abundant biomolecules owing to the so‐called ‘dilution effect’ (Libault et al., 2017). The unique functions of single cells could not be distinguished while making a bulk measurement of tissue. Studying cell phenotypes and behavior becomes imperative to understand developmental dynamics and response to environment in plants (Shulse et al., 2019). Over the last few years, there has been a tremendous technological advancement in terms of new imaging, miniaturization, automation, and microfluidics, thus enabling high‐throughput sequencing of encapsulated single cells (Prakadan et al., 2017). Single‐cell omics thus aim at identifying, quantifying, and characterizing different components of cells including transcriptome (Shulse et al., 2019), proteome (Dai & Chen, 2012; Levy & Slavov, 2018), and metabolome (de Souza et al., 2020) with spatiotemporal resolution. These high‐resolution datasets provide insights on reconstruction of gene‐regulatory and signaling networks driving cellular identity and function (Efremova & Teichmann, 2020; Libault et al., 2017). Furthermore, the data generated from a single cell are incredibly useful for biological modeling and predictions (Stegle et al., 2015). However, in plants, owing to the challenges such as cell heterogeneity, existence of multiple cell states and cell walls, single‐cell studies are limited unlike in animal and microbial cells (Libault et al., 2017). Nevertheless, single cell omics research is slowly gaining momentum with the emerging technological and computational advancement.

### 2.7 Phenomics and high‐throughput phenotyping

Phenomics is the key to exploit the gains of genomics resources. In recent years, crop phenomics has greatly evolved to generate multidimensional phenotypic data at multiple levels from cell level, organ level, plant level to population level (Dhondt et al., 2013; Houle et al., 2010; Lobos et al., 2017). It involves high‐throughput, accurate, and automated measurements of phenotypic information such as plant growth, architecture, and composition at different scales. With the recent technological advances, large‐scale phenotyping data acquisition and processing became possible, which remained a major bottleneck for functional genomics studies and crop breeding (Yang et al., 2020). Phenotypes, such as high‐throughput shoot phenotyping, root phenotyping, canopy, leaf traits, among others, could be measured using high‐throughput phenotyping platforms (Jin et al., 2020). Further sensor technologies now enable detailed recording of the environmental history of plants and, in turn, the dynamic response of crops to the environment. For example, drones or unmanned aerial vehicles, and pocketPlant3D equipped with multiple sensors, such as hyperspectral imaging as well as computed tomography imaging to targeted metabolic sensors, are used to measure traits such as leaf area index estimation, detect weeds and pathogens, and predict yield (Jin et al., 2020; Yang et al., 2020).

Major progress has been made in high‐throughput phenotyping under controlled environments (Pratap et al., 2019). Application of such technology to field conditions are rapidly developing, including vision‐guided robotics (Pieruschka & Schurr, 2019). High‐throughput shoot and root phenotypic data were collected in several model plants and crop species under controlled conditions (Yang et al., 2020). The performance of a plant or crop is affected by multiple genes that interact with multiple environments throughout their growth and development. Advanced sensor, machine vision, and automation technology could now be used to record the crop dynamic response that could further be integrated with the sequence information (Jin et al., 2020). Since phenomics uses several types of sensors simultaneously, data acquisition in a systematic manner is also crucial beginning from experimental set up to data generation and interpretation (Pratap et al., 2019). As the genomes of several model and nonmodel crop species have been sequenced, it is highly required to describe the whole‐crop phenotype. This is important to link gene and QTL to crop phenotypes for dissecting key adaptive traits (Yang et al., 2020).

### 2.8 QTL‐omics

The greatest challenge to the agricultural research community is to be able to correlate and translate gene function to crop improvement in the field under the relevant set of environmental conditions. Predicting complex phenotypic traits from gene networks is complicated by genetic control and environmental effects among different growth and developmental processes of plants (Hammer et al., 2016). To resolve this, multidimensional analysis could provide useful information in understanding genotype–phenotype association, unlike single‐data‐type study designs. Integration of omics approaches leads to QTL mapping and identification of underlying genes. The QTL‐omics will be an integral part, dealing with generation and analysis of large‐scale multiomics data and is broadly defined as characterization of QTL using omics data (Kumawat et al., 2016). However, the biggest challenge is to integrate data from genome sequencing, transcriptomics, proteomics, metabolomics, and phenomics and to make sense of it. Otherwise, QTL‐omics is one of the best approaches to capture the genetic variation present among the whole gene pool for specific quantitative traits in target environment. For example, modeling of QTL has led to the prediction of multigenic traits such as leaf growth and nitrogen accumulation in maize using the systems biology approach (Reymond et al., 2003). Similar efforts were also made in tomato (*Solanum lycopersicum* L.) by integrating expression QTL with metabolite QTL (Schauer et al., 2006). In soybean, QTL‐omics has been applied to characterize mapped QTL. In addition, this approach has also been used in novel QTL mapping and characterization (Kumawat et al., 2016).

All these omics approaches discussed above provide high‐dimensional datasets on different modalities that are only discrete components for a comprehensive view of the plant system. In this regard, systems biology aims to integrate our understanding of how different biological components function to provide insight into the plant system and to develop predictive models on their response when perturbed.

## 3 SYSTEMS BIOLOGY

**FIGURE 2:**
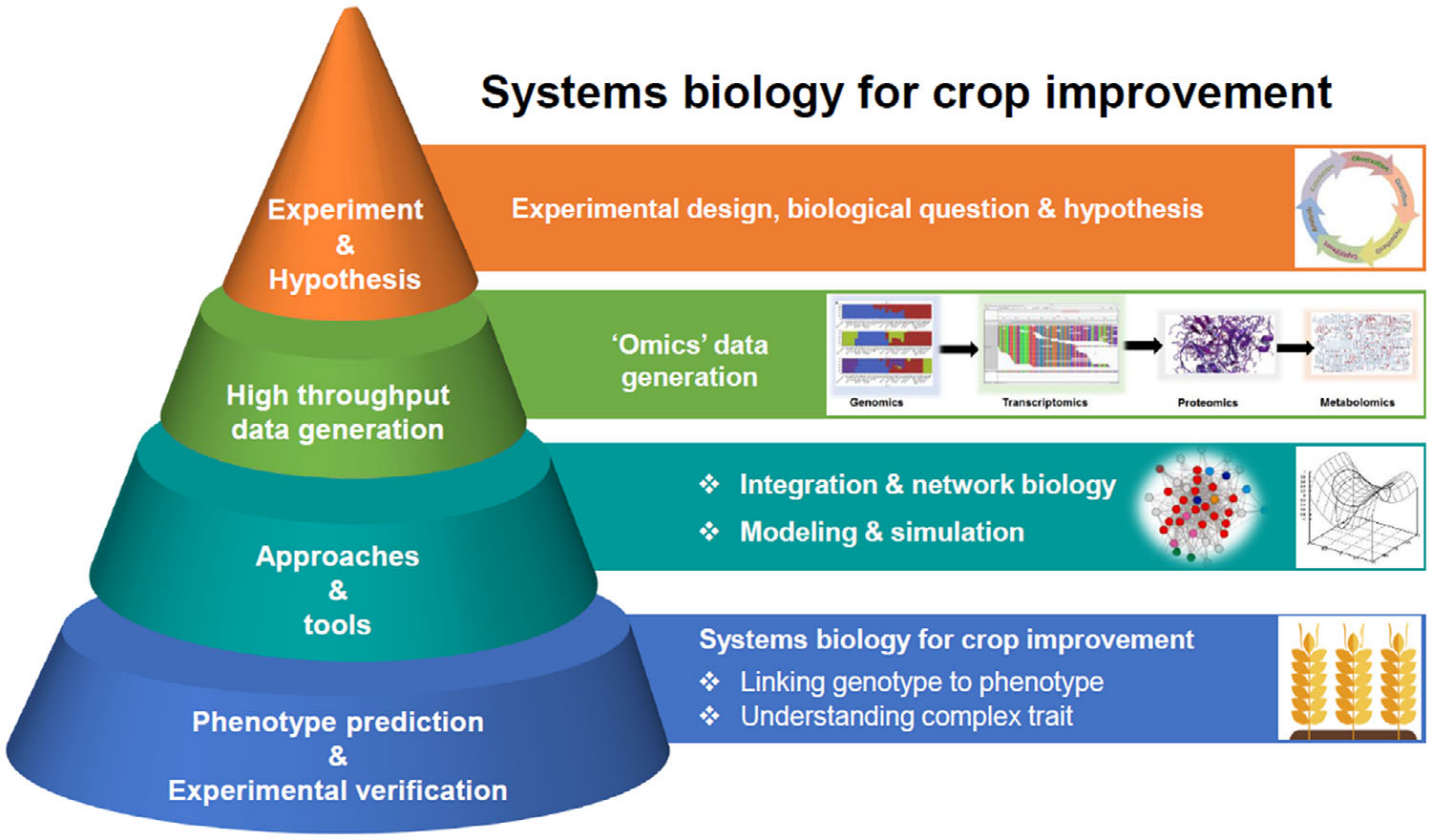
A schematical representation of the systems biology approach for crop improvement. Systems biology provides great potential for sustainable agriculture by understanding complexity of multiple traits bridging the genotype–phenotype gap. It is a strategic approach where it precisely studies the response of each level of biological organization and aims at understanding the complexity of the system as a whole through data integration coupled with mathematical modeling to gain predictive abilities over key agricultural traits

One of the major goals of crop biology research is to maximize yield and reduce losses resulting from various stress factors. As the problem to be tackled is multifaceted, so is the solution multidisciplinary. Understanding the cellular response at each level of the organization has been possible because of the advances in technology that have led to the generation of a huge amount of data from genome, transcriptome, proteome, metabolome, and high‐throughput phenome that is routine now. However, most of the times, these data have been studied independently until recently. Integration of transcriptomics, proteomics, and metabolomics would greatly facilitate identification and dissection of complex plant regulatory networks (Urano et al., 2010). In this regard, systems biology emerged as a promising multidisciplinary research field that integrates large omics datasets coupled with well‐designed mathematical models to confirm hypothesis and predict biological systems (Figure 2; Hong et al., 2019; Sauer et al., 2007). This provides a more holistic understanding of system‐wide response during growth, development, and stress adaptation, critical for next‐generation breeding of climate‐ready crops. However, the first step to a systems biology approach is to devise hypotheses based on prior knowledge. This becomes the basis for a systems biology experimental design and is the most critical step (Pinu et al., 2019). An overview of the various steps involved in a systems biology approach and its application in crop research have been provided in the following sections.

### 3.1 Data integration

The major challenge in integrating omics data remains with the processing, scaling, and analyzing the multidimensional dataset to extract meaningful biological inferences. Integration and analysis of the datasets generated from multiple platforms involve data acquisition, preprocessing, appropriate normalization, and integration into a single matrix. This integrated dataset is generally subjected to multivariate analysis and looked for strong correlations among the biological entities. Genes, proteins, and metabolites with similar patterns were then classified into clusters (Redestig & Costa, 2011; Smilde et al., 2009). Most of the statistical methodologies include dimensionality reduction and studying the coexpressed clusters among the different data measured on the same samples. This is based on the assumption that biological entities showing similar expression patterns across the same samples have hypothetical functional relationships (González et al., 2012). Several platforms to integrate multidimensional omics datasets are available such as mixOmics, OnPLS modeling, Integromics, sparse Multi‐Block Partial Least Squares, and COVAIN (see Misra et al., 2019; Sun & Weckwerth, 2012). In this regard, PANOMICS platform provides integration of complex omics datasets generated from genomics, epigenomics, transcriptomics, proteomics, posttranslational modifications proteomics, metabolomics, and phenomics (Weckwerth et al., 2020). Recently, a systematic multiomics data integration approach, different methodologies, software tools, web applications, and databases for plant systems biology have been proposed (Jamil et al., 2020; Pinu et al., 2019).

These integrative multiomics studies have been used to identify disease mechanisms for improved prognostic and predictive marker identification that reflect molecular pathways in humans (Eddy et al., 2020; Hasin et al., 2017). Similarly, in crop plants, these approaches enable the study of plant metabolism and understand the molecular mechanisms underlying plant phenotypes with potential agronomic importance. Light‐specific metabolic and regulatory signatures were identified using transcriptomics, metabolomics, and genome‐scale in silico modeling in rice (Lakshmanan et al., 2015). Recently, transcriptomics, proteomics, and metabolomics data were analyzed to complement information that provided insights into the fertility transition mechanisms in a pigeonpea thermosensitive male sterile line for its potential use in two‐line hybrid breeding (Pazhamala et al., 2020). In another study, how flavonoid and isoflavonoid metabolism alters in response to ethylene and abscisic acid treatment was studied in soybean leaves by integrating proteomics and metabolomics (Gupta et al., 2018). Table 1 provides a few of the recent studies conducted in crop plants by integrating multiomics data.

**TABLE 1:** Some examples of crop research studies using an integrated omics approach

Study no.	Plant species	Study	Omics approaches	Reference
1	Rice	Plant response to ozone	Transcriptomics, proteomics, metabolomics	Cho et al., 2008
2		Physiological and nutritional quality		Galland et al., 2017
3	Maize	Bt and glyphosate resistant		Barros et al., 2010
4		Carotenoid biosynthesis		Decoutcelle et al., 2015
5		Nitrogen use efficiency		Amiour et al., 2012
6	Soybean	Primary metabolism regulation in response to Rhizoctonia foliar blight disease	Transcriptomics, metabolomics	Copley et al., 2017
7	Common bean	Characterizing variability	Transcriptomics, proteomics, metabolomics	Mensack et al., 2010
8	Tomato	Primary and secondary metabolism		Balcke et al., 2017
9	Sesame	Drought stress	Transcriptomics, metabolomics	You et al., 2019
10	Berry	Drought stress	Transcriptomics, proteomics, metabolomics	Ghan et al., 2015
11	Pepper	Fruit development	Transcriptomics, proteomics	Liu et al., 2019

Furthermore, multiomics data provides a link between phenotype and genome variation to offer new data layers for genomics‐based predictions (Azodi et al., 2020; Weckwerth et al., 2020). In crops such as maize, multiple omics data were integrated into prediction models for improved prediction accuracy (Azodi et al., 2020; Guo et al., 2016; Schrag et al., 2018; Xu et al., 2017). Multiomics data are being increasingly used for phenotypic prediction as it is not restricted to the genome but a result of biological regulation in response to the environment (Acharjee et al., 2016; Li et al., 2019).

### 3.2 Network biology

Cells respond to various genetic and environmental changes through biological processes that are regulated at multiple levels, both transcriptional and translational. Expression of genes are regulated through gene‐to‐gene interaction, epigenetic modification, mutations, transcription factors, and other mechanisms. Most of the plant response and adaptation to stress are specifically controlled by regulatory networks (Gehan et al., 2015; Urano et al., 2010). Reconstruction of pathways and networks using transcriptome, proteome, and metabolome data can help understand these regulatory networks and their functional interaction among the biological entities (Moreno‐Risueno et al., 2010). Interaction among biological entities to carry out cellular functions can be represented as networks and graphs, elucidating biological relationships among genes, proteins, and metabolites (Weckwerth, 2011; Weckwerth et al., 2004). Briefly, omics data are appropriately normalized to obtain a similarity matrix and, subsequently, an adjacency matrix generated is transformed into an undirected graph or a network abstraction. (Langfelder & Horvath, 2008; Langfelder et al., 2013; Redestig & Costa, 2011). Principles, methods, and tools of network inference for exploring biological details, evolutionary origin, and understanding the network structure for predicting biological functions can be found in recent reports (Hao et al., 2016; Lee et al., 2015; Saint‐Antoine & Singh, 2020). Network‐based approaches are limited by current knowledge as well as predicted relationships between biological variables, for instance, Bayesian networks. Several platforms to integrate multidimensional omics datasets are available (Misra et al., 2019). Further, different methodologies, software tools, web applications, and databases for integrating multidimensional omics datasets have been reported (Pinu et al., 2019). 

A system can thus be mathematically represented as a set of nodes linked by edges, where nodes are the biological entity and edges indicate their interaction or relationship, and the highly connected nodes are referred to as hub nodes (Langfelder & Horvath, 2008). Hub nodes provide stronger interactions than the nodes in the periphery of the network module. Biologically, these nodes serve as regulators and could have downstream effects on the pathways (McCormack et al., 2016). Identifying hub nodes have significant implications in detecting key components controlling or affecting simple or complex traits. Thus, networks can provide new biological insights and predict key components and their regulatory influence (Albert, 2007). For this purpose, an experimentally tested genome‐scale rice gene network, RiceNet, was constructed that could accurately be used to predict gene functions in monocotyledonous species (Lee et al., 2011). Further, a barley (*Hordeum vulgare* L.) coexpressed gene network generated using transcriptome data identified gene clusters associated with response to drought stress and biogenesis of cellulose (Mochida et al., 2011). In soybean, a flowering gene network could identify the regulatory roles of *GmCOL1a* and *GmCOL1b* in flowering (Wu et al., 2019). Weighted‐gene coexpression network analysis (Zhang & Horvath, 2005) was used to generate gene coexpression network to identify regulatory networks and key genes controlling seed set and size (Du et al., 2017) and nodulation and nitrogen fixation (Wu et al., 2019) in soybean, whereas pollen fertility and seed set in pigeonpea (Pazhamala et al., 2017) and acquisition of desiccation tolerance in *Boea hygrometrica* (Lin et al., 2019), among many others. On the other hand, the protein–protein interaction network was found useful in investigating complex biological activities and understanding the ways in which external signals are perceived and transduced to trigger specific plant responses (Hao et al., 2016; Struk et al., 2019). In case of Arabidopsis, an *Arabidopsis thaliana* Protein Interactome Database (Cui et al., 2007; Ding & Kihara, 2019) and a dense protein–protein interaction network of plant tricarboxylic acid cycle was generated (Zhang et al., 2017). A protein interaction network associated with salt tolerance in rice was also reported (J. Wang et al., 2013). Furthermore, metabolite–metabolite association networks constructed based on correlation algorithms can comprehensively describe the response of the biological system to environmental perturbation (Jahagirdar & Saccenti, 2020; Jahagirdar et al., 2019; Kose et al., 2001; Rosato et al., 2018; Weckwerth et al., 2004). A study clearly demonstrated the effect of different levels of plant growth regulators and agroecosystem environment on the tomato metabolome using a metabolic network (Fatima et al., 2016). The above‐mentioned network inference studies were within a single omics type; however, several valuable insights were provided through integration across different omics types. Complex network interactions in nitrogen metabolism and signaling in crop plants has been reported using integrated omics approaches (Fukushima & Kusano, 2014). RiceNet was quantitatively integrated with proteomics dataset to predict proteins involved in abiotic stress resistance namely, XA21‐mediated immunity (Lee et al., 2011). In tobacco (*Nicotiana tabacum* L), coexpression gene modules and metabolite modules were integrated to identify gene–metabolite relationship. The study identified key and novel genes and potential regulators of important regulatory networks including carotenoid metabolism pathway (P. Liu et al., 2020). Similarly, Mounet et al. (2009) integrated transcriptome and metabolome data to identify subsets of genes involved in fruit development and metabolism in tomato.

In brief, global gene coexpression networks has been found to be a promising approach for studying and high‐throughput prediction of specialized metabolite pathways. Thus, network biology can transform our understanding of the genetic basis of how plants respond and cope with the changing environment (Wisecaver et al., 2017). To be able to do so, it is important to have an appropriate temporal design for the acquisition of omics data that needs to be analyzed with a network biology approach. Network biology is potentially a powerful tool for modeling the cellular response to adverse environmental perturbations (McCormack et al., 2016).

### 3.3 Systems modeling

The next step in network biology is the dynamic modeling that allows a comprehensive view of the gene expression shaping protein behaviors in a way to elicit metabolites in response to external triggers in plants (McCormack et al., 2016). Systems modeling primarily tests biological hypotheses and further extended to predict a system‐wide response. To make a biological sense of the data, computational modeling, including dynamic simulation models and machine learning approaches, is used. In simple words, the biological system is simulated based on the hypothesis devised by the biologist, which is tested over the course of time. The simulated results are then compared with the experimental data to evaluate the hypothesis and further refined to match the experiments. This is critical to achieving a perfect prediction of the system's behavior under dynamic conditions and time (Macklin, 2019; Muthuramalingam et al., 2019).

Four major approaches have been suggested for cellular modeling: constraint‐based modeling (Price et al., 2004), metabolic modeling (Steuer et al., 2006), analysis of metabolic control (Reder, 1988), and cybernetic modeling (Kompala et al., 1984). Constraint‐based modeling requires information regarding metabolic reaction and flux capacity and can also perform with low experimental data. This method has clear advantage over all other methods and has been used to reconstruct metabolic networks at the whole‐cell or genome scale, which is an emerging application together with omics data to increase the predictive power (Sroka et al., 2011). This approach has been found to provide deep insights into the cellular metabolism in response to abiotic stress in rice (Muthuramalingam et al., 2019; Raman & Chandra, 2009). Contrastingly, metabolic modeling corresponds to a specific component of metabolism using equations that speculate the dynamic changes in the concentration of metabolites. In plants, metabolic modeling remains limited, as it requires kinetic variables to model networks at large scale, which is not available for most of the biochemical reactions (Muthuramalingam et al., 2019). Analysis of metabolic control, on the other hand, is one of the most widely used tools for the study of control in plants, which quantifies the response of system variables (e.g. fluxes) to small changes in system parameters (e.g. the amount or activity of the individual enzymes). The cybernetic modeling framework was originally used for macroscopic input–output models describing substrate uptake, growth, and product formation. Later, this approach has been extended for application to more complex, intracellular metabolism. The software applications and mark‐up languages available to facilitate systems modeling include Cell Illustrator (Nagasaki et al., 2010), COBRAToolbox (Schellenberger et al., 2011), Acorn (Sroka et al., 2011), CellNetanalyzer (Klamt et al., 2007), among many others (see Muthuramalingam et al., 2019; Pinu et al., 2019).

Machine learning uses applied statistical and computational techniques to teach machines to extract patterns in the data including features and labels for predictive modeling (Camacho et al., 2018). Generally, there are two types of machine‐learning methods: supervised and unsupervised learning (McMurray & Hollich, 2009). Supervised learning includes regression algorithm and classification algorithms techniques (random forest, multivariate regression), which are used to predict the outcome with labeled data. Unsupervised learning, on the other hand, includes clustering algorithms and association rule learning, employed for clustering data, detecting outliers, and dimensionality reduction (Mishra et al., 2019). The analytics consist of data preprocessing, modeling, and active learning to deal with the complexity and intricacies of omics data. Machine learning has emerged as a powerful toolbox for integration of high‐dimensional biological data, extracting systems‐level insights and predicting a range of outcome (Gazestani & Lewis, 2019; van Dijk et al., 2020). In plant research, phenotype prediction and understanding genotype‐to‐phenotype relationship remains the fundamental goal. Predicting plant phenotypes, for example, stress response and quality traits from the multiomics data is highly required for crop improvement. In this regard, a study in maize classified DNA sequence regions into active vs. (inactive) pseudogenes, using features such as DNA methylation (Sartor et al., 2019). Another study reported identification of key genes, proteins, and metabolites that are predictive of potato (*Solanum tuberosum* L.) tuber quality from transcriptomics, proteomics, and metabolomics data. This study used random forest regression for integrating the omics data (Acharjee et al., 2016). In plant research, prediction using ensemble of neural networks is quite popular. The prediction performance of phenotype depends on the predictive modeling and analytics and is an exhaustive subject that has been reviewed recently (Kim & Tagkopoulos, 2018; Tong et al., 2020).

## 4 CROP IMPROVEMENT APPLICATIONS

Systems biology provides great potential for sustainable agriculture by understanding the complexity of multiple traits bridging the genotype–phenotype gap. It can be used to model and analyze multigenic traits linked with agricultural productivity such as plant architecture, nitrogen use efficiency, water use efficiency, and abiotic and biotic stress tolerance. Plant genetics and molecular biology have been extensively contributing to the plant breeding programs. However, because of the upsurge in recent high‐throughput experimental analyses and computational power, there is a transformative possibility to integrate multiple disciplines to explain any given complex trait. The availability of whole‐genome sequence information, generation of omics datasets with rapidly advancing technologies, analytical tools, and software (Table 2) have made it possible to study and address abiotic stress‐responsive cellular, biochemical and molecular mechanisms as well as signaling processes. For instance, well‐designed mathematical models based on time series datasets allow the identification of key candidate genes for potential use in the breeding programs and thus devise a systems biology‐based breeding strategy (Lavarenne et al., 2018). Furthermore, they convincingly provided a roadmap to use systems biology for future breeding programs. They inferred that the ultimate practical use of systems biology is to unravel the complex interactions governing the multigenic trait for crop improvement. Development of comprehensive models by integrating multiomics data with high throughput and precise phenotyping will ensure efficient breeding programs to improve agronomically important and complex traits in the future. This would be critical to developing future‐ready crops that can sustain and increase productivity even in marginal environments faced with biotic and abiotic stresses (Gehan et al., 2015). We anticipate the use of systems biology for devising and utilizing novel breeding approaches for crop improvement in the future.

**TABLE 2:** A list of key tools and databases for systems biology research

Tools	Description	Link	References
Biochemical, Genetic and Genomic (BiGG) knowledge base	Reconstruction of biochemical networks; Genome‐scale metabolic models (GEMs)	http://bigg.ucsd.edu	Feist et al., 2009; King et al., 2016
Systems biology markup language (SBML)	A medium for representation and exchange of biochemical network models	http://www.sbml.org/	Hucka et al., 2003
JSBML 1.0	Provides a smorgasbord of options to encode systems biology models	http://sbml.org/Software/JSBML	Rodriguez et al., 2015
VirtualPlant	A software platform to support systems biology research	http://virtualplant.bio.nyu.edu	Katari et al., 2010
Babelomics	A complete suite of web tools for functional analysis of genome‐scale experiments	http://www.babelomics.org.	Al‐Shahrour et al., 2006
Pathway tools version 13.0	Integrated software for pathway and genome informatics and systems biology	http://BioCyc.org/download.shtml	Karp et al., 2010
TEPIC 2	A framework for fast, accurate and versatile prediction, and analysis of TF binding from epigenetics data	https://github.com/SchulzLab/TEPIC	Schmidt et al., 2019
Mergeomics	Multidimensional data integration to identify pathogenic perturbations to biological systems	Available in Bioconductor	Shu et al., 2016
Crops in silico	An integrative platform for plant systems biology research	http://cropsinsilico.org/	Zhu et al., 2016
AtPID (*Arabidopsis thaliana* protein interactome database)	An integrative platform for plant systems biology	http://atpid.biosino.org/	Cui et al., 2007
MGS	Interaction‐based simulations for integrative spatial systems biology	http://mgs.spatial‐computing.org/	–
VESPER 1.5	Spatial prediction software for precision agriculture	–	Whelan et al., 2002
MetExplore	Visualization of metabolites in the context of the whole network/reactions	http://metexplore.toulouse.inra.fr/joomla3/index.php	Cottret et al., 2010
ProMeTra	Visualization and combining datasets from transcriptomics, proteomics, and metabolomics	https://omictools.com/prometra‐tool	Neuweger et al., 2009
KaPPA‐View	Integrates transcriptomics and metabolomics data to map pathways	http://kpv.kazusa.or.jp/kappa‐view/	Tokimatsu et al., 2005

## 5 CHALLENGES AND PROSPECTS

The use of multiple omics techniques (i.e., genomics, transcriptomics, proteomics, metabolomics, epigenomics, and single‐cell omics) is becoming increasingly popular in plant sciences as sequencing cost drops and expertise rises. As a result, sequencing and resequencing information that is generated for several crops enabled the identification of novel alleles from diverse sources irrespective of the availability of the genome sequence. Furthermore, rapid advances in omics technologies provide an opportunity to generate new and informative datasets in different plant species. Integration of these genomes and functional omics data with genetic and phenotypic information in an efficient manner would lead to the identification of genes and pathways responsible for important agronomic traits. On the other hand, the huge amount of data generated on the functional components of the cell are still underutilized even in a model crop like rice (Muthuramalingam et al., 2019). Moreover, the growth and productivity of crops in the field conditions are hindered not by individual stress but a combinatorial effect of multiple stresses, both biotic and abiotic. Hence, there is an urgent need to direct future research toward the discovery of key players, molecular networks, and models that could unravel the complex cellular and molecular functions to enhance agronomic traits in crops. In this regard, systems biology offers huge potential in crop research to revolutionize our understanding on how plants respond to growth and environmental constraints (Muthuramalingam et al., 2019).

Systems biology and integrating omics approach provides a more inclusive molecular perspective of plant biology than individual approaches. However, the integrated approach of multiple omics platforms remains an ongoing challenge because of their inherent data differences. The collection of accurate multiomics data on different molecular and functional components is most critical to systems modeling and prediction, for which there are no standard protocols shared among the global community (Kim & Tagkopoulos, 2018; Macklin, 2019). In addition, model‐based integration is often restricted to well‐defined model organisms (Pinu et al., 2019). Constant evolution of databases and data analysis tools would help meaningful biological interpretation of multiomics data. Various technical challenges during experimentation, data generation, integrations, handling, sharing, and interpretation are comprehensively described in Misra et al. (2019) and Macklin (2019). Hence, it is high time to form a highly connected community for plant systems biology research, instead of isolated efforts, and to develop and share resources, databases, and software tools. This is even more crucial because of the urgency to double crop yields by 2050 under uncertain climate scenarios.

## CONFLICT OF INTEREST

The authors declare no conflicts of interest.
